# Genomic Investigation of Disseminated Gonococcal Infections, Minnesota, USA, 2024

**DOI:** 10.3201/eid3110.250785

**Published:** 2025-10

**Authors:** Daniel Evans, Hannah Friedlander, Khalid Bo-Subait, Jefferson Dennis, John Kaiyalethe, Bradley Craft, Matthew Plumb, Bonnie Weber, Laura Bohnker-Voels, Kelly Pung, Alyssa Mondelli, Jacob Garfin, Jennifer Zipprich, Paula Snippes-Vagnone, Kathryn Como-Sabetti, Ruth Lynfield

**Affiliations:** Minnesota Department of Health, St. Paul, Minnesota, USA

**Keywords:** Sexually transmitted infections, genomic surveillance, *Neisseria gonorrhoeae*, bacterial infections, bacteria, Minnesota, United States

## Abstract

This article summarizes a genomic investigation of a 4-fold increase in disseminated gonococcal infections in Minnesota, USA, in 2024. We detected the emergence of a *Neisseria gonorrhoeae* strain of a rarely observed sequence type, which carries a *porB1a* allele previously associated with disseminated disease and lacks a gonococcal genetic island.

Although most *Neisseria gonorrhoeae* infections remain localized to the urogenital tract of infected patients, this sexually transmitted pathogen can spread to other body sites and cause systemic infections (*1*). Disseminated gonococcal infections (DGIs) are thought to occur in <3% of infections, often cause severe illness, and are poorly understood in their pathogenesis (*1*,*2*).

In Minnesota, USA, the Minnesota Department of Health (MDH) performs routine surveillance of urogenital and disseminated *N. gonorrhoeae* infections, according to state reporting rules that include submission of isolates or other clinical materials for gonococcal infections in normally sterile sites. In this article, we report an increase in DGI cases in Minnesota in 2024. We used whole-genome sequencing (WGS) technology to investigate that increase. Our objectives were to assess the relatedness of DGI-causing strains and to identify potential genetic factors that contributed to dissemination.

## The Study

In 2024, a total of 27 culture-confirmed *N. gonorrhoeae* infections from normally sterile sites were reported to MDH, a nearly 4-fold increase over the average yearly number of cases reported since surveillance began in 2020. Because further investigation of those cases was conducted as public health surveillance subject to state reporting rules, institutional review board approval was not necessary. We confirmed the species of 23 isolates from 20 DGI cases (n = 2 cases with multiple isolates collected) by using Biotyper matrix-assisted laser desorption/ionization time-of-flight mass spectrometry (Bruker, https://www.bruker.com) with an internally validated research use only database. We prepared WGS libraries from those isolates by using the Illumina DNA Prep kit (Illumina, https://www.illumina.com) and sequenced genomes on the Illumina MiSeq platform with reagent kit v2 (500 cycles) or the Illumina NextSeq 2000 platform with P1 reagents (300 cycles). We assembled and phylogenetically compared genomes by using the Spriggan v1.3.0 (*3*) and Dryad v3.0.0 (*4*) bacterial bioinformatics pipelines (Appendix). To classify assembled genomes by sequence type (ST) and resolve gonococcal genetic island sequences, we queried assembled genomes against the PubMLST database (*5**,**6*). Then, we performed *N. gonorrhoeae* multilocus sequence typing (MLST) by antimicrobial resistance (NG-STAR) by using the NG-STAR v2.0 database and identified *porB* allele types (Appendix Table 1) (*7*).

**Figure 1:**
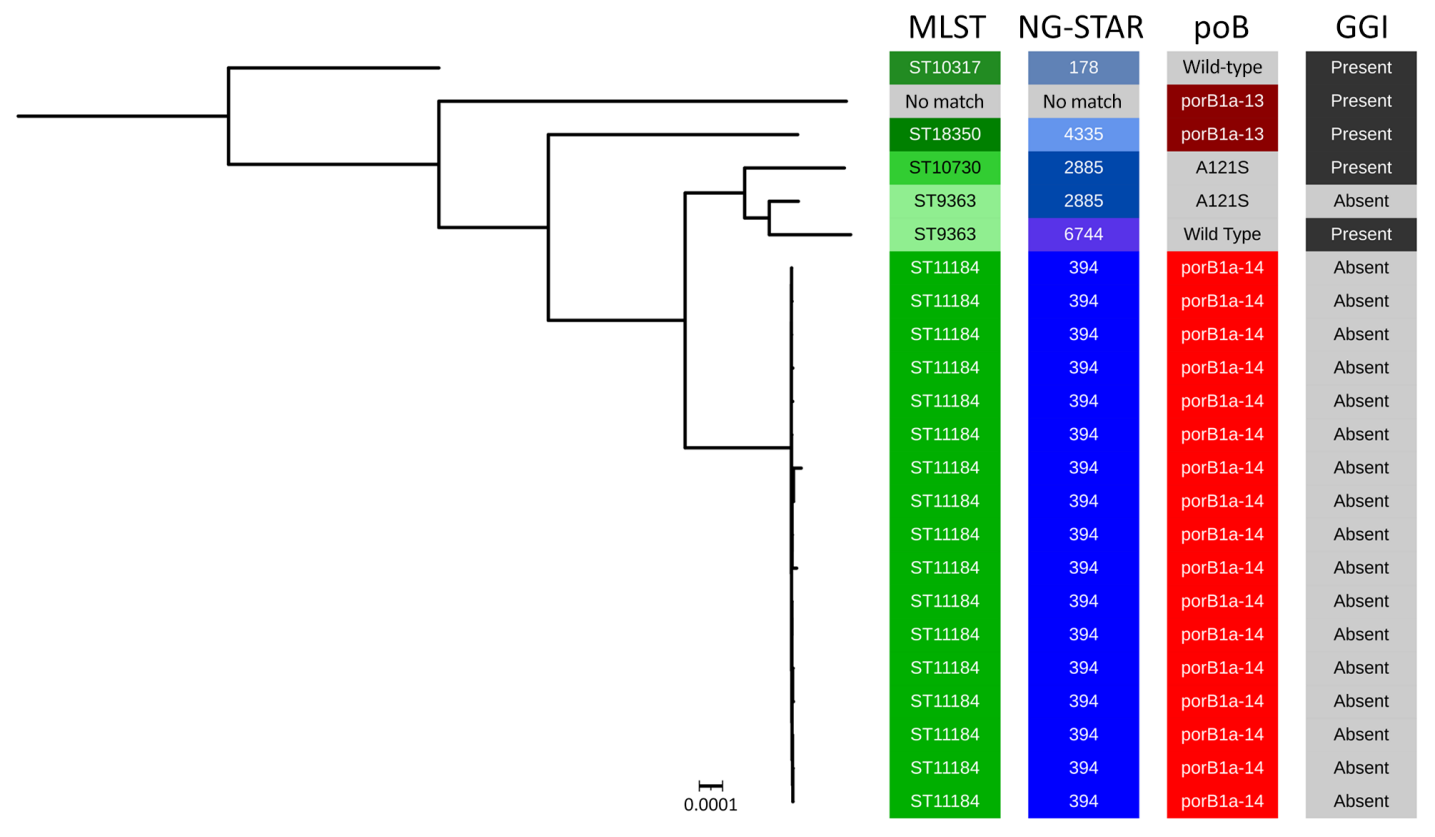
Maximum-likelihood, distance-scaled, core gene phylogenetic tree of *Neisseria gonorrhoeae* genomes from disseminated gonococcal infection cases in Minnesota, 2024. We constructed this tree by using the Dryad v3.0 pipeline (Appendix) (*4*). GGI, presence or absence of a gonococcal genetic island sequence; MLST, multilocus sequence typing; NG-STAR, *N. gonorrhoeae* sequence type by antimicrobial resistance; porB, porin B allele type within the NG-STAR classification scheme; ST, sequence type.

**Figure 2:**
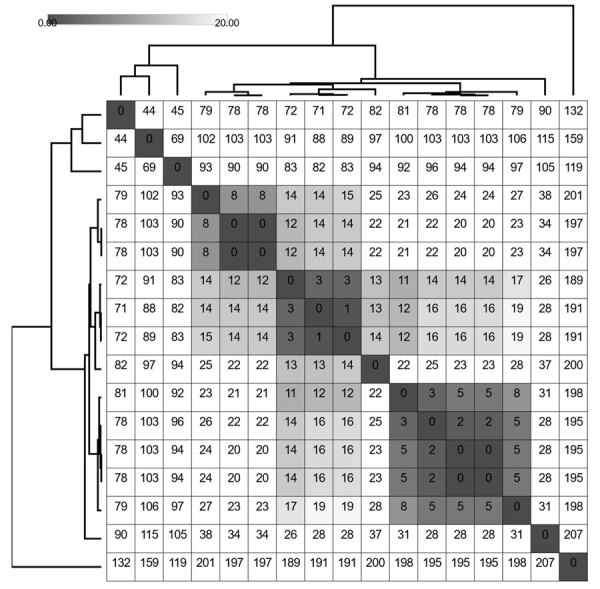
Pairwise single-nucleotide polymorphism (SNP) matrix of *Neisseria gonorrhoeae* sequence type (ST) 11184 genomes from disseminated gonococcal infection cases, Minnesota, USA. We clustered and visualized the matrix by using Morpheus (https://morpheussoftware.net) from SNPs identified within a reference-based genome alignment we generated by using the Dryad v3.0 pipeline (*4*), with an internal reference genome. The grayscale shading represents SNP values of 0–20. The branches represent hierarchical clustering of rows and columns of pairwise SNP calls.

Of the 20 DGI cases with available WGS data, we classified 14 (70.0%) isolates as both ST11184 and NG-STAR profile 394. We sequenced 17 genomes of that type, which included 3 repeat isolates from 2 patients (Figure 1). One genome did not identically match any documented MLST profile or NG-STAR profile for *N. gonorrhoeae*. The ST11184 genomes ranged in genetic similarity from 0–207 pairwise single-nucleotide polymorphisms (SNPs) by the reference-based approach in the Dryad pipeline (Figure 2) (*4*). Repeating that analysis by using Bakta v1.9.4, Panaroo v1.5.0, and snp-dists v0.8.2 in a reference-free, core genome alignment-based approach yielded a range of 4–168 pairwise SNPs (Appendix Figure 1) (*8**–**12*).

All genomes belonging to this closely related ST11184 strain encoded a *porB1a* allele sequence (NG-STAR *porB* type 14). Two other genomes, including the genome without an MLST match, carried a *porB1a* allele that matched NG-STAR *porB* type 13. Previous studies have associated strains encoding those *porB1a* alleles with an increased likelihood of causing disseminated infection (*13*). PubMLST queries also revealed that the ST11184 genomes did not carry a gonococcal genetic island, which functions in horizontal transfer of antimicrobial resistance and virulence genes are well documented (*14*).

To contextualize the genomes of our DGI-causing strains, we used PubMLST and the National Center for Biotechnology Information Pathogen Detection Browser (PDB; https://www.ncbi.nlm.nih.gov/pathogens/isolates) tool to search for publicly available genomes that were closely related to those we sequenced. After performing comparisons with >60,000 *N. gonorrhoeae* genomes that were publicly available as of May 2025, metadata from which indicated that most were from isolates collected during 2015–2024, the PDB tool grouped all 17 Minnesota ST11184 genomes into their own cluster (cluster identification no. PDS000214546.1). When those genomes were first processed by PDB, the cluster included only 1 other genome belonging to an isolate collected from a urogenital infection in Minnesota in September 2024 and sequenced through the Centers for Disease Control and Prevention’s Gonococcal Isolate Surveillance Project, and no other genomes in the database (*15*). All other DGI genomes were grouped into other PDB clusters, except for the genome that did not match any MLST or NG-STAR profile (Appendix Table 1).

**Figure 3:**
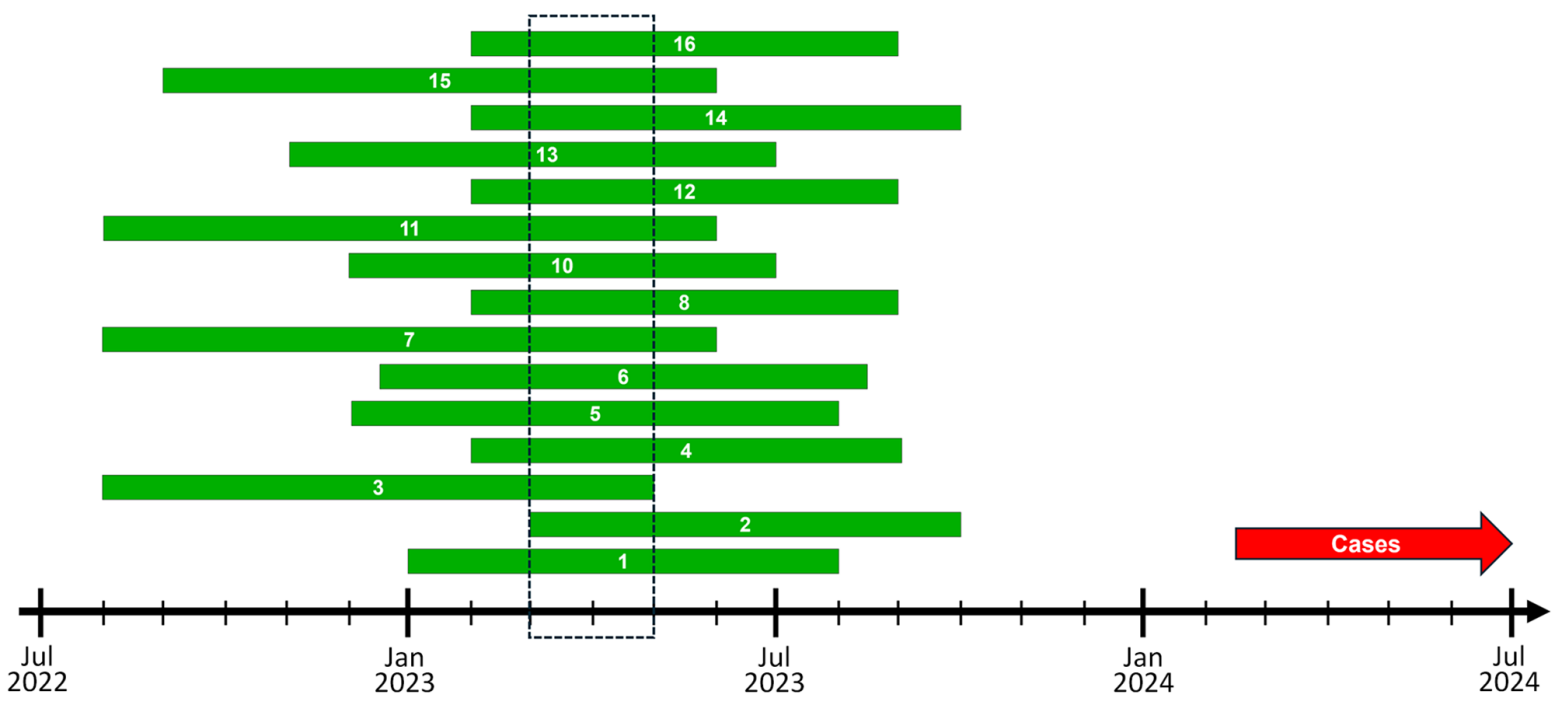
Phylodynamic inference of the emergence of *Neisseria gonorrhoeae* sequence type (ST) 11184 genomes that infected disseminated gonococcal infection cases, Minnesota, USA. The green bars denote the 90% CIs of the estimated time to most recent common ancestor (tMRCA) of a clade of 18 ST11184 genomes from Minnesota, rounded to the nearest month. We calculated tMRCAs by refining a maximum-likelihood, distance-scaled, core gene phylogenetic tree of publicly available ST11184 genomes into a time-scaled tree by using TreeTime v0.11.4 software (Appendix) (*10*). The dashed box denotes the period in which the tMRCA 90% CIs of all 16 iterations of time-scaled refinement overlapped. The red arrow denotes the time after the earliest collection of an isolate from a 2024 disseminated gonococcal infection case in Minnesota.

To estimate when the DGI-causing ST11184 strain might have emerged, we generated a core-genome alignment and phylogenetic tree of the Minnesota and publicly available ST11184 genomes by using Bakta, Panaroo, and IQTree2 v2.3.6 (*8**–**11*). We then used TimeTree v0.11.4 to perform 16 time-scaled phylodynamic analyses with iteratively varied inputs (*11*) (Appendix). The dataset used for this analysis consisted of 41 genomes, including all 17 ST11184 genomes from Minnesota, the previously published genome from the same PDB cluster, all publicly accessible genomes documented on PubMLST as belonging to that MLST (n = 10), and all genomes shown by the National Center for Biotechnology Information PDB tool to be closely related to the PubMLST genomes (n = 13). Of the 24 publicly available genomes from other sources, only 5 (20.8%) genomes were assigned to NG-STAR profile 394. We used the distance-scaled tree as an input for phylodynamic analysis, which grouped all 18 Minnesota ST11184 genomes from the same PDB cluster into their own subclade (Appendix Figure 2). Across all 16 iterations, TreeTime calculated estimated times of most recent common ancestors of that clade during December 2022–June 2023, and 90% confidence intervals all intersected during March–April 2023 (Figure 3; Appendix Table 2).

Epidemiologists at MDH completed investigations of all DGI infections with the ST11184 strain (n = 14). Among those cases, the median patient age was 40.5 years (range 28–60 years). Eight (57.1%) case-patients had *N. gonorrhoeae* isolated from joint or synovial fluid and 6 (42.9%) from blood samples. Thirteen (92.9%) case-patients were hospitalized, with a mean length of stay of 4.7 days (range 2–15 days), and no patients died. Six (42.9%) case-patients reported underlying conditions, including diabetes (n = 2 [14.3%]), concomitant sexually transmitted infection (n = 2 [14.3%]), immunosuppressive therapy (n = 1 [7.1%]), and history of intravenous drug use within the previous 12 months (n = 1 [7.1%]). Three (21.4%) case-patients reported being on HIV preexposure prophylaxis medications. Case-patients predominantly resided in Hennepin and Ramsey counties, within the Minneapolis-St. Paul metropolitan area (n = 13 [92.9%]). We did not identify any direct epidemiologic links between cases from those investigations.

## Conclusions

The genomic data from this study of DGI cases in Minnesota, USA, yielded several conclusions and opened further lines of investigation. The similarity among the genomes of the ST11184 *N. gonorrhoeae* strain indicated that it emerged in Minnesota recently, and its substantial difference from other previously documented genomes from global surveillance suggests it might not have been observed elsewhere. The presence of 2 *porB1a* alleles among DGI-causing isolates also reinforces previous studies that associated the allele with disseminated infections (*2*,*13*). Conversely, the breadth of genetic distance between the genomes of the emerging strain contraindicates potential direct transmission among all infected cases and raises questions about the incidence of unobserved transmission through urogenital infections. Future genomic surveillance of DGI is critical to expand comparisons of strains causing disseminated versus urogenital infections, identify other genetic determinants of dissemination, retrospectively sequence other known DGI-causing isolates from previous years, and further evaluate the utility of phylodynamic methods in tracking gonococcal outbreaks.

Appendix Additional information about genomic investigation of disseminated gonococcal infections, Minnesota, USA, 2024.
