# Supplementary material for: Genomic Investigation of Disseminated Gonococcal Infections, Minnesota, USA, 2024
Source: Emerg Infect Dis. 2025 Oct;31(10):2003–7. doi: 10.3201/eid3110.250785 (PMC12483114; doi:10.3201/eid3110.250785)
Supplement: Appendix — Additional information about genomic investigation of disseminated gonococcal infections, Minnesota, USA, 2024. [file 25-0785-Techapp-s1.pdf]

Article DOI: https://doi.org/10.3201/eid3110.250785

EID cannot ensure accessibility for supplementary materials supplied by authors. Readers who have difficulty accessing supplementary content should contact the authors for assistance.

Genomic Investigation of Disseminated Gonococcal Infections, Minnesota, USA, 2024

Appendix

Appendix Methods

Genome assembly, quality assessment, and phylogenetic comparison

We performed genome assembly, and quality assessment using the Spriggan v1.3.0 bioinformatics pipeline (*1*,*2*). Spriggan incorporates the following tools for trimming of low-quality sequencing reads, genome assembly, assessment of genome quality and coverage, classification of MLST, and detection of contamination: BBtools v38.76, FastQC v0.11.8, Shovill v1.1.0, QUAST v5.0.2, BWA-MEM v0.7.17-r1188, samtools v1.10, Kraken2 v2.0.8, Pandas v1.3.2, and MultiQC v1.11 (*3–10*) (Li H, et al. preprint, https://arxiv.org/abs/1303.3997).We used the Dryad v3.0.0 pipeline to perform pairwise single nucleotide polymorphism (SNP) comparisons among *N. gonorrhoeae* genomes (*10*). Dryad incorporates the following tools for genome assembly quality assessment, genome alignment, and SNP calling: QUAST v5.0.2, Kraken2 v2.0.8, Prokka v1.14.5, Roary v3.12.0, and CFSAN SNP Pipeline v2.0.2 (*6*,*8*,*12–14*).

Phylodynamic analysis using TreeTime software

We used TreeTime v0.11.4 to estimate the time of the most recent common ancestor (tMRCA) of the Minnesota ST11184 genome cluster (*15*). We used Bakta v1.9.4, Panaroo v1.5.0, and IQTree2 v2.3.6 to construct core genome alignments and maximum likelihood phylogenetic trees of all ST11184 genomes from Minnesota and publicly available on sequencing data repositories (n = 41 genomes) (*16–18*). We performed 16 iterations of this analysis, each of which differed by any of four input parameters. The first parameter was the core gene filtering mode used by Panaroo to construct a core genome alignment. This was

performed using either the "strict" mode, which filters out any potential contaminant genes present in fewer than 5% of analyzed genomes, or "sensitive" mode, which does not delete any potential contaminant genes based on quality or low prevalence. The second was whether TreeTime was executed using an input tree of the phylogenetic tree generated by Bakta, Panaroo, and IQTree2, or of the optimized phylogenetic tree generated when estimating a molecular clock rate by the "treetime clock" command. The third was whether the evolutionary clock rate for tMRCA calculation was estimated by TreeTime under default settings, or whether a fixed clock rate was pre-calculated using the "treetime clock" command and then used as inputs ("–clock-rate" and "–clock-std-dev" flags). The fourth was whether the estimated or pre-set clock rates were calculated using all genomes in the input tree and alignment, or whether the molecular clock rate model excluded any genomes whose residuals in the least-square regression of root-to-dip versus inferred date exceeded three interquartile ranges (IQRs) of the regression's distribution.

All phylodynamic analyses were performed using commands to perform stochastic resolution of polytomies ("–stochastic-resolve" flag), account for covariance within the input phylogeny ("–covariation" flag), and calculate temporal divergence with 90% confidence intervals using the default marginal maximum-likelihood method ("–confidence" flag). For the 24 ST11184 genomes from public databases, we input specimen collection dates to the highest precision as they were publicly documented. Of those genomes, 7 (29.2%) had collection dates reported to the day, 6 (25.0%) were reported to the month, 9 (37.5%) were reported to the year, and 2 (8.3%) had no publicly reported specimen collection dates.

Appendix Table 1. Summary of WGS metadata and NCBI public repository reference numbers for Minnesota DGI genomes*

NCBI BioProject Number	NCBI BioSample Number	Sequence Read Archive (SRA) ID Number	Pathogen Detection Browser (PDB) Cluster Number	MLST Profile	NG-STAR Profile	Porin B (NG-STAR)	GGI Presence
PRJNA1204341	SAMN46038853	SRR31856393	PDS000213116.4	ST10730	2885	3	Present
PRJNA1204341	SAMN46038854	SRR31856392	PDS000214546.1	ST11184	394	14	Absent
PRJNA1204341	SAMN46038855	SRR31856381	PDS000214546.1	ST11184	394	14	Absent
PRJNA1204341	SAMN46038856	SRR31856377	PDS000174403.2	ST18350	4335	13	Present
PRJNA1204341	SAMN46038857	SRR31856376	PDS000009126.47	ST10317	178	1	Present
PRJNA1204341	SAMN46038858	SRR31856375	PDS000214546.1	ST11184	394	14	Absent
PRJNA1204341	SAMN46038859	SRR31856374	PDS000214546.1	ST11184	394	14	Absent
PRJNA1204341	SAMN46038860	SRR31856373	PDS000214546.1	ST11184	394	14	Absent
PRJNA1204341	SAMN46038861	SRR31856372	PDS000214546.1	ST11184	394	14	Absent
PRJNA1204341	SAMN46038862	SRR31856371	PDS000214546.1	ST11184	394	14	Absent
PRJNA1204341	SAMN46038863	SRR31856391	PDS000214546.1	ST11184	394	14	Absent
PRJNA1204341	SAMN46038864	SRR31856390	PDS000214546.1	ST11184	394	14	Absent
PRJNA1204341	SAMN46038865	SRR31856389	PDS000102532.19	ST9363	2885	3	Absent
PRJNA1204341	SAMN46038866	SRR31856388	No match	No match	No match	13	Present
PRJNA1204341	SAMN46038867	SRR31856387	PDS000214546.1	ST11184	394	14	Absent
PRJNA1204341	SAMN46038868	SRR31856386	PDS000214546.1	ST11184	394	14	Absent
PRJNA1204341	SAMN46038869	SRR31856385	PDS000046008.27	ST9363	6744	100	Present
PRJNA1204341	SAMN46038870	SRR31856384	PDS000214546.1	ST11184	394	14	Absent
PRJNA1204341	SAMN46038871	SRR31856383	PDS000214546.1	ST11184	394	14	Absent
PRJNA1204341	SAMN46038872	SRR31856382	PDS000214546.1	ST11184	394	14	Absent
PRJNA1204341	SAMN46038873	SRR31856380	PDS000214546.1	ST11184	394	14	Absent
PRJNA1204341	SAMN46038874	SRR31856379	PDS000214546.1	ST11184	394	14	Absent
PRJNA1204341	SAMN46038875	SRR31856378	PDS000214546.1	ST11184	394	14	Absent

*"MLST" = multilocus sequence type; "NG-STAR" = N. gonorrhoeae sequence typing by antimicrobial resistance (NG-STAR); "Porin B" = porB allele type as defined within the NG-STAR scheme; "GGI Presence" = presence or absence of a gonococcal genetic island sequence.

Appendix Table 2. Summary of input settings to calculate estimated times of most recent common ancestor (tMRCAs) for the Minnesota-specific clade of *N. gonorrhoeae* ST11184 genomes by 16 iterations of time-scaled phylodynamic analysis, using TreeTime v0.11.4 (*15*)*

Input Alignment	Input Tree	Clock Rate Calculation	Clock Rate Inclusion	Clock Rate (mutations/site/year)	Estimated Clade tMRCA (90% Confidence Interval)
Strict (filtered)	Distance-scaled IQTree2	TreeTime default	All genomes (n = 41)	9.8E-06 ±7.0E-07	February 2023 to August 2023
Strict (filtered)	Distance-scaled IQTree2	TreeTime default	3 IQR limit (n = 39)	6.7E-06 ±6.6E-07	August 2022 to May 2023
Strict (filtered)	Distance-scaled IQTree2	TreeTime pre-calculated	All genomes (n = 41)	1.00E-05	February 2023 to August 2023
Strict (filtered)	Distance-scaled IQTree2	TreeTime pre-calculated	3 IQR limit (n = 37)	8.60E-06	December 2022 to June 2023
Strict (filtered)	Optimized TreeTime	TreeTime default	All genomes (n = 41)	9.6E-06 ±7.0E-07	February 2023 to August 2023
Strict (filtered)	Optimized TreeTime	TreeTime default	3 IQR limit (n = 39)	6.9E-06 ±6.6E-07	September 2022 to May 2023
Strict (filtered)	Optimized TreeTime	TreeTime pre-calculated	All genomes (n = 41)	1.00E-05	February 2023 to September 2023
Strict (filtered)	Optimized TreeTime	TreeTime pre-calculated	3 IQR limit (n = 37)	8.60E-06	November 2022 to June 2023
Sensitive (no-filter)	Distance-scaled IQTree2	TreeTime default	All genomes (n = 41)	9.6E-06 ±7.0E-07	February 2023 to August 2023
Sensitive (no-filter)	Distance-scaled IQTree2	TreeTime default	3 IQR limit (n = 39)	6.5E-06 ±6.6E-07	August 2022 to May 2023
Sensitive (no-filter)	Distance-scaled IQTree2	TreeTime pre-calculated	All genomes (n = 41)	1.02E-05	March 2023 to September 2023
Sensitive (no-filter)	Distance-scaled IQTree2	TreeTime pre-calculated	3 IQR limit (n = 37)	8.61E-06	January 2023 to July 2023
Sensitive (no-filter)	Optimized TreeTime	TreeTime default	All genomes (n = 41)	9.2E-06 ±7.0E-06	February 2023 to August 2023
Sensitive (no-filter)	Optimized TreeTime	TreeTime default	3 IQR limit (n = 39)	6.8E-06 ±6.6E-07	August 2022 to April 2023
Sensitive (no-filter)	Optimized TreeTime	TreeTime pre-calculated	All genomes (n = 41)	1.02E-05	March 2023 to September 2023
Sensitive (no-filter)	Optimized TreeTime	TreeTime pre-calculated	3 IQR limit (n = 37)	8.61E-06	December 2022 to July 2023

*"Input Alignment" = method used to generate a core gene alignment; "Input Tree" = use of the maximum likelihood phylogenetic tree generated by IQTree2 v2.3.6, or of the TreeTime-optimized version of the IQTree2 output; "Clock Rate Calculation" = use of TreeTime's default settings to calculate evolutionary clock rates under default settings, or of the output of a pre-calculation step; "Clock Rate Inclusion" = the inclusion of all genomes in the alignment and tree when calculating clock rates, or excluding those whose inferred mutation rates exceed 3 IQRs of the regression model.

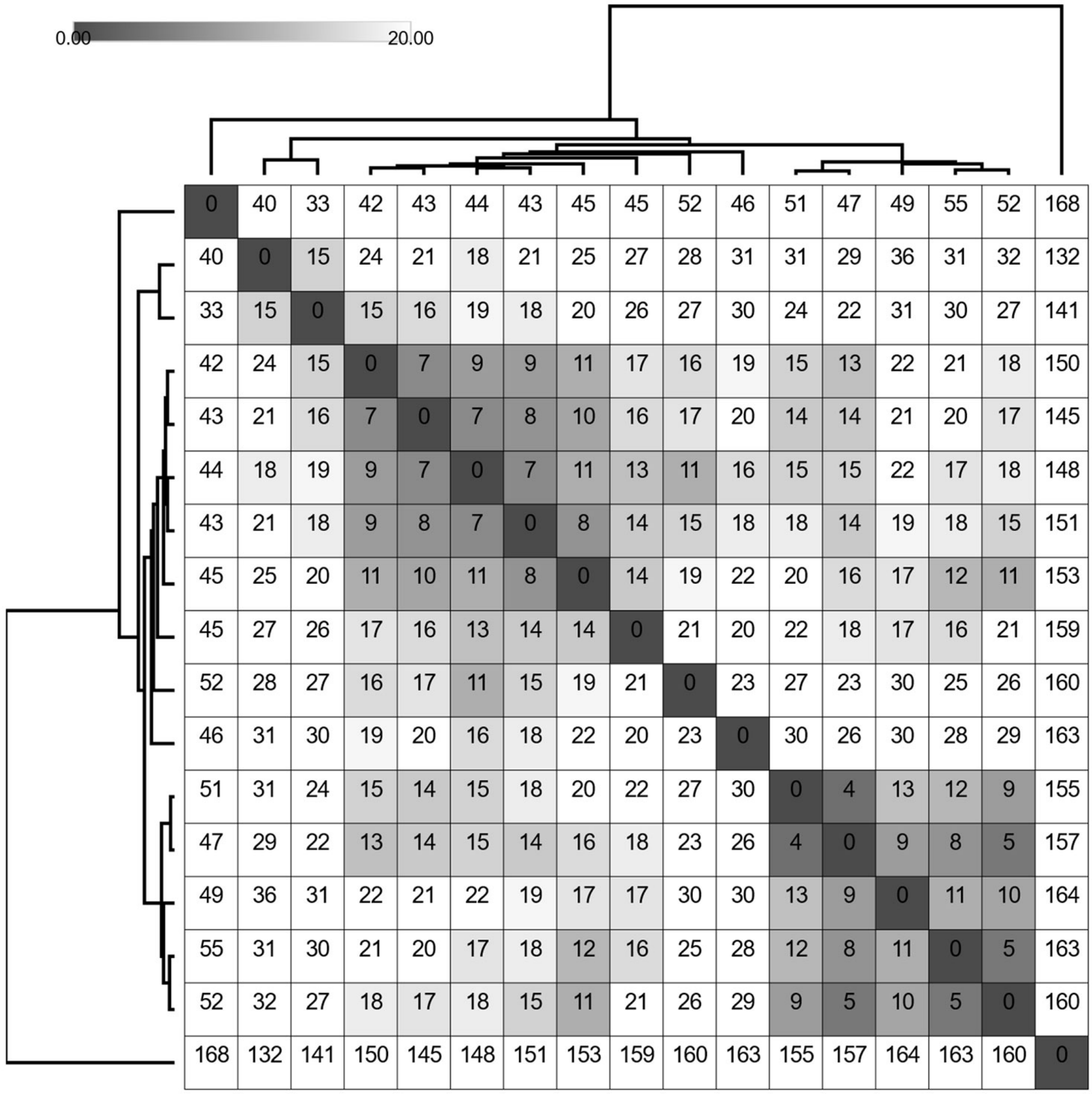

Appendix Figure 1. Supplemental single nucleotide polymorphism (SNP) matrix of *N. gonorrhoeae* ST11184 genomes from Minnesota DGI cases. The matrix was clustered and visualized using Morpheus software (Broad Institute) from SNPs identified within a reference-free core genome alignment generated by Panaroo v1.5.0 from genomes annotated by Bakta v1.9.2 (*16*).

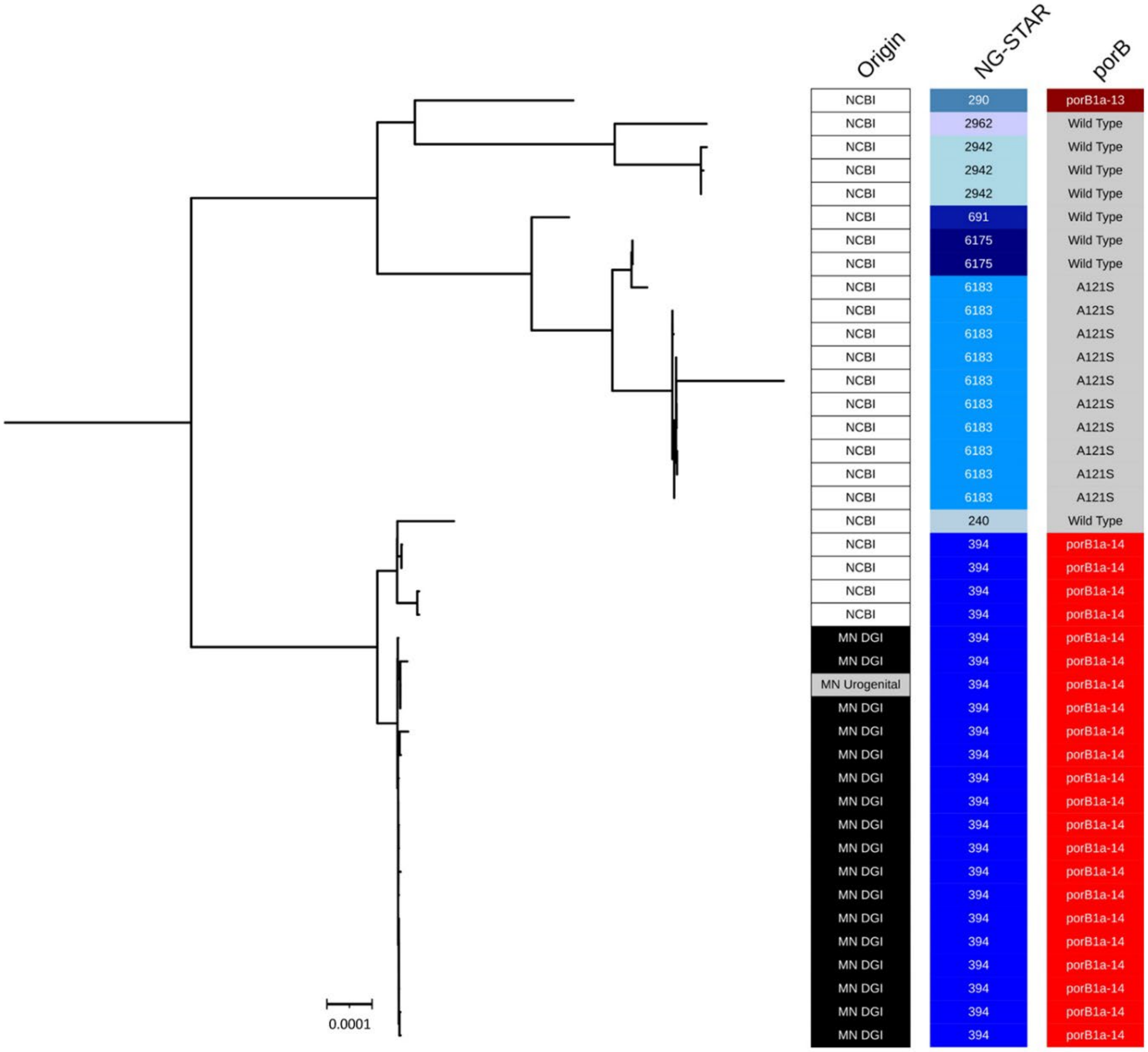

Appendix Figure 2. Maximum likelihood, reference-free, core gene phylogenetic tree of *N. gonorrhoeae* ST11184 genomes from publicly accessible databases. This tree was used as an input for time-scaled phylodynamic refinement as described in the main text and Appendix Methods. Annotations from left to right: "Origin" = genome from a Minnesota DGI case, a Minnesota urogenital gonorrhea case, or a contextual non-Minnesota genome from a publicly accessible database; "NG-STAR" = *N. gonorrhoeae* sequence type by antimicrobial resistance; "porB" = porin B allele type within the NG-STAR classification scheme.

References

1. Wisconsin State Laboratory of Hygiene. *Spriggan*. 2022 [cited 2025 May 27]. https://github.com/wslh-bio/spriggan

2. Ewels PA, Peltzer A, Fillinger S, Patel H, Alneberg J, Wilm A, et al. The nf-core framework for community-curated bioinformatics pipelines. Nat Biotechnol. 2020;38:276–8. PubMed https://doi.org/10.1038/s41587-020-0439-x

3. Bushnell B. BBMap. 2015 [cited 2025 May 27]. https://sourceforge.net/projects/bbmap

4. Andrews S. FastQC: a quality control tool for high throughput sequence data. 2010 [cited 2025 May 27]. https://github.com/s-andrews/FastQC

5. Seemann T. Shovill: faster SPAdes assembly of illumina reads. 2017 [cited 2025 May 27]. https://github.com/tseemann/shovill

6. Mikheenko A, Prjibelski A, Saveliev V, Antipov D, Gurevich A. Versatile genome assembly evaluation with QUAST-LG. Bioinformatics. 2018;34:i142–50. PubMed https://doi.org/10.1093/bioinformatics/bty266

7. Danecek P, Bonfield JK, Liddle J, Marshall J, Ohan V, Pollard MO, et al. Twelve years of SAMtools and BCFtools. Gigascience. 2021;10:giab008. PubMed https://doi.org/10.1093/gigascience/giab008

8. Wood DE, Lu J, Langmead B. Improved metagenomic analysis with Kraken 2. Genome Biol. 2019;20:257. PubMed https://doi.org/10.1186/s13059-019-1891-0

9. Reback J, Mendel JB, McKinney W, Van den Bossche J, Augspurger T, Cloud P, et al. Pandas 1.3.2. 2021 [cited 2025 May 27]. https://ui.adsabs.harvard.edu/abs/2021zndo...5203279R/abstract

10. Ewels P, Magnusson M, Lundin S, Käller M. MultiQC: summarize analysis results for multiple tools and samples in a single report. Bioinformatics. 2016;32:3047–8. PubMed https://doi.org/10.1093/bioinformatics/btw354

11. Wisconsin State Laboratory of Hygiene. *Dryad*. 2023 [cited 2025 May 27]. https://github.com/wslh-bio/dryad.

12. Seemann T. Prokka: rapid prokaryotic genome annotation. Bioinformatics. 2014;30:2068–9. PubMed https://doi.org/10.1093/bioinformatics/btu153

13. Page AJ, Cummins CA, Hunt M, Wong VK, Reuter S, Holden MT, et al. Roary: rapid large-scale prokaryote pan genome analysis. Bioinformatics. 2015;31:3691–3. PubMed https://doi.org/10.1093/bioinformatics/btv421

14. Davis S, Pettengill JB, Luo Y, Payne J, Shpuntoff A, Rand H, et al. CFSAN SNP Pipeline: an automated method for constructing SNP matrices from next-generation sequence data. PeerJ Comput Sci. 2015;1:e20. https://doi.org/10.7717/peerj-cs.20

15. Sagulenko P, Puller V, Neher RA. TreeTime: Maximum-likelihood phylodynamic analysis. Virus Evol. 2018;4:vex042. PubMed https://doi.org/10.1093/ve/vex042

16. Schwengers O, Jelonek L, Dieckmann MA, Beyvers S, Blom J, Goesmann A. Bakta: rapid and standardized annotation of bacterial genomes via alignment-free sequence identification. Microb Genom. 2021;7:000685. PubMed https://doi.org/10.1099/mgen.0.000685

17. Tonkin-Hill G, MacAlasdair N, Ruis C, Weimann A, Horesh G, Lees JA, et al. Producing polished prokaryotic pangenomes with the Panaroo pipeline. Genome Biol. 2020;21:180. PubMed https://doi.org/10.1186/s13059-020-02090-4

18. Minh BQ, Schmidt HA, Chernomor O, Schrempf D, Woodhams MD, von Haeseler A, et al. IQ-TREE 2: new models and efficient methods for phylogenetic inference in the genomic era. Mol Biol Evol. 2020;37:1530–4. PubMed https://doi.org/10.1093/molbev/msaa015
